# Comparison of responses of human melanoma cell lines to MEK and BRAF inhibitors

**DOI:** 10.3389/fgene.2013.00066

**Published:** 2013-05-08

**Authors:** Clare J. Stones, Ji Eun Kim, Wayne R. Joseph, Euphemia Leung, Elaine S. Marshall, Graeme J. Finlay, Andrew N. Shelling, Bruce C. Baguley

**Affiliations:** ^1^Department of Obstetrics and Gynaecology, The University of AucklandAuckland, New Zealand; ^2^Auckland Cancer Society Research Centre, The University of AucklandAuckland, New Zealand

**Keywords:** mitogen-activated protein kinase pathway, melanoma treatment, NRAS, BRAF, MEK, ERK, vemurafenib, trametinib

## Abstract

The *NRAS* and *BRAF* genes are frequently mutated in melanoma, suggesting that the NRAS-BRAF-MEK-ERK signaling pathway is an important target for therapy. Two classes of drugs, one targeting activated BRAF and one targeting MEK, are currently undergoing clinical evaluation. We have analysed the *NRAS* and *BRAF* mutational status of a series of 44 early passage lines developed from New Zealand patients with metastatic melanoma. 41% of the lines analysed had *BRAF* mutations, 23% had *NRAS* mutations, and 36% had neither. We then determined IC_50_ values (drug concentrations for 50% growth inhibition) for CI-1040, a commonly used inhibitor of MEK kinase; trametinib, a clinical agent targeting MEK kinase; and vemurafenib, an inhibitor of mutant BRAF kinase. Cell lines with activating *BRAF* mutations were significantly more sensitive to vemurafenib than lines with *NRAS* mutations or lines lacking either mutation (*p* < 0.001). IC_50_ values for CI-1040 and trametinib were strongly correlated (*r* = 0.98) with trametinib showing ~100-fold greater potency. Cell lines sensitive to vemurafenib were also sensitive to CI-1040 and trametinib, but there was no relationship between IC_50_ values and *NRAS* mutation status. A small number of lines lacking a *BRAF* mutation were sensitive to CI-1040 but resistant to vemurafenib. We used western blotting to investigate the effect on ERK phosphorylation of CI-1040 in four lines, of vemurafenib in two lines and of trametinib in two lines. The results support the view that MEK inhibitors might be combined with BRAF inhibitors in the treatment of melanomas with activated *BRAF*. The high sensitivity to trametinib of some lines with wildtype *BRAF* status also suggests that MEK inhibitors could have a therapeutic effect against some melanomas as single agents.

## Introduction

Malignant melanoma is an important public health issue, particularly in Australia and New Zealand where the incidence rates for melanoma are very high (Coory et al., 2006; Liang et al., 2010). While early stage melanoma can usually be treated successfully by surgery, metastatic melanoma has a poor survival rate and is highly resistant to conventional cytotoxic chemotherapy. Activating mutations in the *BRAF* gene have been reported in 40–70% of melanomas and activating mutations in the *NRAS* gene in another 10–30% (Davies et al., 2002). There is considerable interest in developing therapies targeting this pathway, and clinical trials of drugs such as vemurafenib (PLX4032), which target mutant BRAF protein, have provided very promising results with 81% of patients with *BRAF* mutant melanoma having clinical responses in a Phase I trial (Flaherty et al., 2010). Since preclinical studies indicate that BRAF inhibitors are ineffective in melanomas lacking *BRAF* mutations and may even enhance growth (Hatzivassiliou et al., 2012), advanced clinical trials of vemurafenib and other BRAF inhibitors are being carried out specifically in patients whose melanomas contain *BRAF* mutations (Solit et al., 2006; Flaherty et al., 2010).

Resistance to BRAF inhibitors develops relatively rapidly because of BRAF-independent activation of MEK and ERK (Johannessen et al., 2010) and other chemotherapeutic approaches will be necessary, both for melanomas lacking mutant *BRAF* and for melanomas that have developed resistance. The MEK protein, which functions downstream from BRAF, is thus a further potential target (Johannessen et al., 2010). Preclinical studies with MEK inhibitors reported that *BRAF* mutant melanoma cells growing both *in vitro* and *in vivo* as xenografts were more responsive to MEK inhibition than cell lines with wild type *BRAF* status (Davies et al., 2002; Solit et al., 2006). Furthermore, the new MEK inhibitor trametinib (GSK1120212) has shown evidence of clinical efficacy against melanoma (Falchook et al., 2012), and has shown survival benefits in phase III trial (Flaherty et al., 2012).

In this study, we have characterized the *BRAF* and *NRAS* mutation status of a series of melanoma cell lines developed from New Zealand patients with metastatic melanoma (Marshall et al., 1994; Charters et al., 2011; Kim et al., 2012). We determined the IC_50_ values of these cell lines to CI-1040, a MEK inhibitor that has been utilized extensively in preclinical studies (Sebolt-Leopold, 2004) and compared these values to those for the mutant BRAF inhibitor vemurafenib. For a subset of cell lines we determined IC_50_ values for trametinib. Since rapid development of resistance (within hours) through up-regulation of MEK pathway signaling in the absence of *BRAF* mutations has been reported in melanoma cell lines (Friday et al., 2008), we have also measured in some cell lines the time-dependent effects of CI-1040 and vemurafenib on ERK phosphorylation.

## Materials and methods

### Cell lines and tissue culture

New Zealand Melanoma (NZM) cell lines were derived from metastatic tumors and developed at the Auckland Cancer Society Research Centre, New Zealand. The cell lines were maintained in α-MEM medium (Invitrogen), supplemented with 5% foetal calf serum (Invitrogen), penicillin-streptomycin sulfate, and insulin-transferrin-selenite, in a 37°C incubator at 5% CO_2_ and O_2_. The final concentrations of the supplements in media were 100 units/mL penicillin G, 100 μg/mL streptomycin sulfate, 5 μg/mL insulin, 5 μg/mL transferrin, and 5 ng/mL sodium selenite.

### Genomic profiling of cell lines

DNA from cell lines was sequenced for activating mutations in *NRAS* exon 2 and 3 and *BRAF* exon 11 and 15. DNA was extracted using phenol-chloroform-isoamyl alcohol. Exons of interest were amplified by PCR using Taq polymerase from Qiagen. The primer sequences for *BRAF* exon 15 and *NRAS* exon 2 and 3 were designed using DNA Star; the sequences are provided in Table 1. The primers for *BRAF* exon 11 are from a published source (Davies et al., 2002). The PCR conditions were as follows: an initial denaturation step at 95°C for 5 min, followed by 30 cycles (*BRAF* exon 11) or 40 cycles (*BRAF* exon 15, *NRAS* exon 2 and 3) consisting of denaturation at 95°C for 1 min, annealing at the appropriate temperature for 1 min, extension at 72°C for 1 min, followed by a final extension step at 72°C for 10 min. The annealing temperatures for the PCR reactions were as follows: 60°C for *BRAF* exon 11, 56°C for *BRAF* exon 15, 58°C for *NRAS* exon 2, and 60°C for *NRAS* exon 3. Polyethylene glycol precipitation (Lis and Schleif, 1975) was used to purify the *NRAS* exon 2 and 3 and *BRAF* exon 15 PCR products. Enzymatic digestion of unused PCR reaction ingredients by exonuclease 1 Affymetrix USB and shrimp alkaline phosphatase Affymetrix USB was used to purify *BRAF* exon 11.

**Table 1:** **BRAF and NRAS sequencing primers**.

**Gene and exon**	**Primers**	**Primer sequence**	**Amplicon size**	**Location on reference sequence**
BRAF exon 11	Forward	Davies et al. (2002)	271 bp	140481587-140481567
	Reverse	Davies et al. (2002)		140481275-140481298
BRAF exon 15	Forward	CACCTCATCCTAACACATTTCAAG	765 bp	140453433-140453410
	Reverse	TTTCAACAGGGTACACAGAACAT		140452668-140452690
NRAS exon 2	Forward	ATTAATCCGGTGTTTTTGCGTTCT	633 bp	115258944-115258921
	Reverse	CATCTCTGAATCCTTTATCTCCAT		115258311-115258334
NRAS exon 3	Forward	AACAGCACAAATAAAACAGTCCAG	799 bp	115256971-115256948
	Reverse	GGTTCCAAGTCATTCCCAGTA		115256172-115256192

The reference sequences cited are NC_000007.13 (BRAF) and NC_000001.10 (NRAS).

The PCR products were sequenced using thermal cycle sequencing, with Big Dye Terminator 3.1 chemistry (Applied Biosystems). The sequencing cycle conditions were as follows: an initial denaturation step at 95°C for 5 min followed by 25 amplification cycles of 1 min each of denaturation at 95°C, annealing at 50°C for 5 min, and primer extension at 60°C for 4 min. The sequencing products were purified by ethanol precipitation and the sequences run on an Applied Biosystems 3130XL capillary sequencing machine at the Centre for Genomics and Proteomics, University of Auckland. Mutations were confirmed by sequencing in the opposite direction using separately amplified DNA.

### Determination of IC_50_ values

The sensitivity of the cell lines to inhibitors was measured using a ^3^H-thymidine incorporation method (Marshall et al., 1992). Melanoma cells were plated in 96 well plates at 1000 cells per well and incubated overnight at 37°C at 5% CO_2_ and O_2_. Drugs were added and plates incubated for 5 days at 37°C at 5% CO_2_ and O_2_. ^3^H-thymidine (0.04 μCi/well), 5-fluorodeoxyuridine (0.1 μM), and thymidine (0.1 μM) were added 6 h before harvesting the cultures. To harvest, Pronase (2 mg/mL in 4 mM EDTA in PBS) was added per well for 1 h and the plates incubated at 37°C at 5% CO_2_ and O_2_, to detach the cells. The cells were transferred onto Wallac glass fiber filter mats using a Tomtec cell harvester, and the beta emission counted using a Wallac Trilux Microbeta scintillation counter. IC_50_ values (mean and SEM) were calculated using SigmaPlot.

### Western blot analysis

Cells were plated in 6 well tissue culture plates (Falcon) at 2.5 × 10^5^ cells per well and incubated overnight at 37°C at 5% O_2_ to allow the cells to attach. Drugs were added to the wells on the following day and the cells were harvested at the indicated time points using a lysis buffer containing phosphatase and protease inhibitors (Cheng et al., 2004). The protein concentration of cell lysates was determined using the bicinchoninic acid (BCA) assay and the lysates (50 μ g of protein per well) were subjected to western blotting. The proteins were transferred to PVDF membranes and probed with antibodies for p-ERK, total ERK, p-MEK, total MEK, p-AKT, total AKT, cyclin D1 (all from Cell Signaling Technology), tubulin (Sigma) and β-actin (Millipore). The western blots were photographed using a LAS3000 Luminescent Image Analyzer (Fuji), and quantified using Image J software.

## Results

### *BRAF* and *NRAS* mutations in melanoma cell lines

Screening results for the 44 melanoma cell lines are shown in Table 2. Thirteen lines (30%) had activating V600E and another 2 lines (5%) had activating V600K mutations. The NZM28 line contained a L584F amino acid substitution as well as a G469A substitution, the NZM41 line contained a D594N mutation, and the NZM37 had a Thr600ins mutation. The cell lines were also evaluated for mutations of the *NRAS* gene; four lines (9%) had a Q61K mutation, one a G12D mutation, one a G13L mutation, two a Q61H mutation, and one a Q61R mutation. All the identified mutations are described in the Welcome Trust COSMIC DNA mutation database.

**Table 2:** **Genetic and IC_50_ data for NZM cell lines**.

**Cell line**	**BRAF status**	**BRAF DNA**	**NRAS status**	**NRAS DNA**	**CI-1040 IC_50_ (nM)**	**Trametinib IC_50_ (nM)**	**Vemurafenib IC_50_ (nM)**
NZM1	wildtype	WT	wildtype	WT	<7.8		1600
NZM2	wildtype	WT	wildtype	WT	8.7	0.48	150
NZM3	V600E	GTG to GAG 600	wildtype	WT	36		29
NZM4	V600E	GTG to GAG 600	wildtype	WT	33	0.36	17
NZM5	wildtype	WT	wildtype	WT	16	0.84	255
NZM6	V600E	GTG to GAG 600	wildtype	WT	65		59
NZM7	V600E	GTG to GAG 600	wildtype	WT	38	0.85	33
NZM9	wildtype	WT	wildtype	WT	72		1600
NZM10	wildtype	WT	Q61K	CAA to AAA 61	23	0.63	2500
NZM11	V600E	GTG to GAG 600	wildtype	WT	120		15
NZM13	wildtype	WT	wildtype	WT	1070		
NZM14	V600K	GTG to AAG 600	wildtype	WT	10	0.33	85
NZM15	wildtype	WT	Q61K	CAA to AAA 61	<7.8		1050
NZM17	wildtype	WT	Q61K	CAA to AAA 61	430		2000
NZM19	wildtype	WT	wildtype	WT	102		1600
NZM20	V600E	GTG to GAG 600	wildtype	WT	9.1	0.30	13
NZM21	wildtype	WT	wildtype	WT	101	0.75	
NZM22	wildtype	WT	wildtype	WT	1410	10	1030
NZM23	wildtype	WT	wildtype	WT	740		1040
NZM24	wildtype	WT	G12D	GGT to GAT 12	21		760
NZM28	G469A L584F	GGA to GCA 469 CTT to TTT 584	wildtype	WT	8.6		3.3
NZM29	wildtype	WT	wildtype	WT	710		900
NZM30	V600E	GTG to GAG 600	wildtype	WT	22	0.35	66
NZM31	V600E	GTG to GAG 600	wildtype	WT	17		47
NZM33	wildtype	WT	Q61R	CAA to CGA 61	<7.8	0.36	2300
NZM34	V600E	GTG to GAG 600	wildtype	WT	64		72
NZM35	wildtype	WT	wildtype	WT	520	2.3	1040
NZM36	wildtype	WT	wildtype	WT	8.5		2000
NZM37	Ins T600	Ins ACA 600	wildtype	WT	19		400
NZM38	V600E	GTG to GAG 600	wildtype	WT	99		55
NZM39	wildtype	WT	wildtype	WT	<7.8	0.35	1300
NZM40	wildtype	WT	Q61H	CAA to CAT 61	790	5.5	590
NZM41	D594N	TGA to TAA 594	wildtype	WT	200		660
NZM43	V600K	GTG to AAG 600	wildtype	WT	<7.8		170
NZM44	wildtype	WT	wildtype	WT	140		2000
NZM45	wildtype	WT	Q61L	CAA to CTA 61	170		510
NZM46	wildtype	WT	Q61H	CAA to CAT 61	10		140
NZM48	wildtype	WT	Q61K	CAA to AAA 61	34		550
NZM49	V600E	GTG to GAG 600	wildtype	WT	70	0.40	70
NZM55	V600E	GTG to GAG 600	wildtype	WT	28		3.8
NZM56	wildtype	WT	wildtype	WT	90	1.0	590
NZM58	V600E	GTG to GAG 600	wildtype	WT	67	0.33	25
NZM61	wildtype	WT	wildtype	WT	90	0.75	560
NZM63	wildtype	WT	G13L	GGT to CGT 13	<7.8	0.31	920

### Sensitivity of melanoma lines to CI-1040, vemurafenib and trametinib

**Figure 1:**
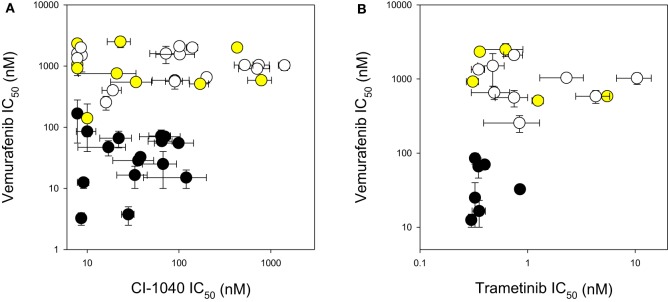
**Comparison of IC_50_ values for (A) CI-1040 vs. vemurafenib and (B) trametinib vs. vemurafenib using a panel of melanoma cell lines.** Black circles: mutant BRAF. Yellow circles: mutant NRAS. White circles: wildtype for BRAF and NRAS. Vertical and horizontal bars indicate the standard errors of the means where available; IC_50_ values of <7.8 nM are shown as 7.8 nM.

The response of the melanoma cell lines to the MEK and BRAF inhibitors was tested using IC_50_ assays and the results are shown in Table 2 and Figure 1. The main study, with CI-1040 (Figure 1A), showed a clustering of low IC_50_ values for CI-1040 and vemurafenib for cell lines with activating *BRAF* mutations (V600E and V600K). The NZM28 cell line, which contained both G469A and L584F substitutions was very sensitive to both inhibitors and thus fell into this cluster. On the other hand NZM37, with a Thr600 insertion, and NZM41, with a D594N substitution, were relatively insensitive to vemurafenib (Table 2). Lines with *NRAS* mutations (Q61K, G12D, Q61H, and Q61R) were all resistant to vemurafenib and there was no correlation between the presence of mutation and sensitivity to CI-1040. A smaller study (Figure 1B) compared cell line sensitivity to trametinib. IC_50_ values for trametinib were highly correlated with those for CI-1040 (*r* = 0.985) but trametinib was, on average, more than 100-fold more potent. Clustering of IC_50_ values was again observed, with all vemurafenib sensitive lines also showing sensitivity to trametinib.

### Modulation of ERK phosphorylation in response to MEK and BRAF inhibitors

**Figure 2:**
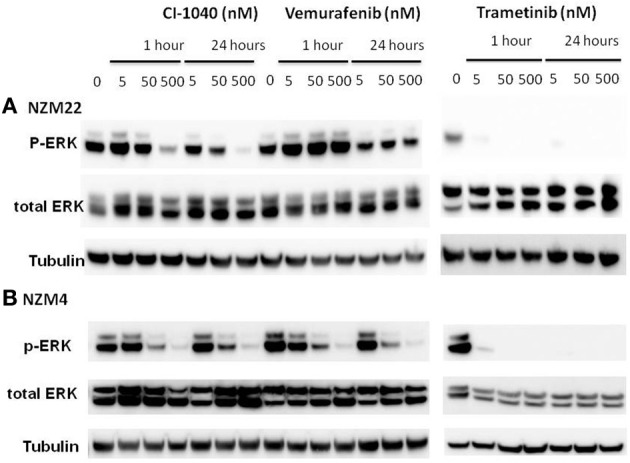
**Western blots showing changes in ERK phosphorylation 1 and 24 h after addition of different concentrations of the MEK inhibitors CI-1040 and trametinib, and the mutant BRAF inhibitor vemurafenib. (A)** NZM22 line (BRAF wild type). **(B)** NZM4 line (V600E BRAF).

In order to compare signaling changes in the ERK pathway to inhibition of proliferation, we measured changes to ERK phosphorylation induced by CI-1040, trametinib, and vemurafenib in NZM22, which is *NRAS* and *BRAF* wildtype and relatively resistant to all three inhibitors (Table 2), and in NZM4, which is BRAF mutant and relatively sensitive to the three inhibitors tested. ERK phosphorylation was more sensitive in NZM4 cells than in NZM22 cells in response to both CI-1040 and vemurafenib at both the 1-h and 24-h time points (Figure 2). Comparison of sensitivity to trametinib was also carried out but both cell lines were sensitive to the lowest drug concentration used.

**Figure 3:**
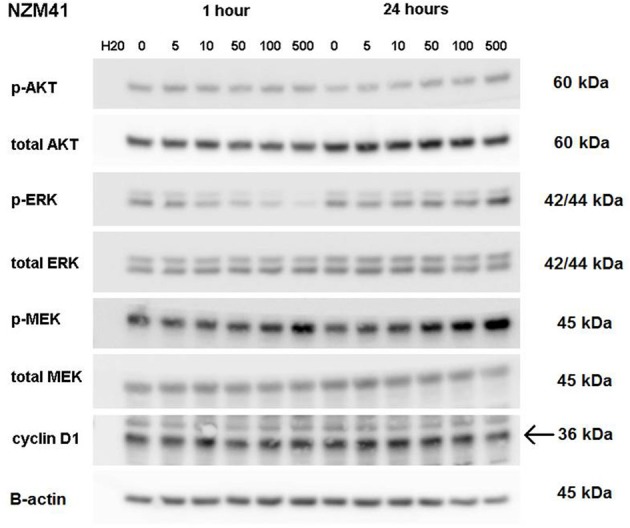
**Western blots showing pathway signaling in response to CI-1040 (nanomolar concentrations) for the NZM41 line (BRAF D549N mutation) at 1 and 24 h.** The arrow indicates the protein of interest in blots where non-specific bands are also present.

**Figure 4:**
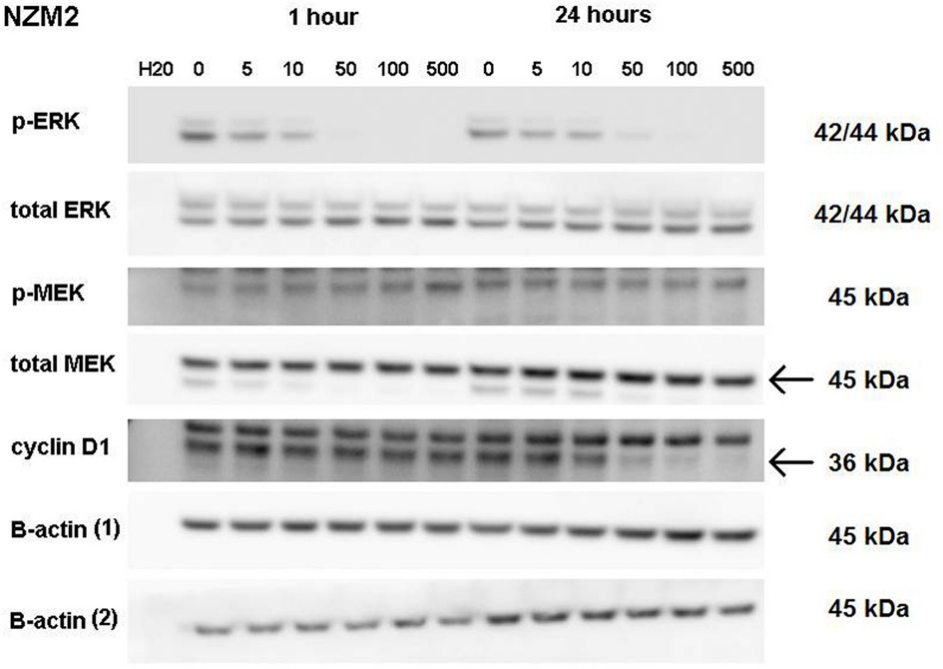
**Western blots showing pathway signaling in response to CI-1040 (nanomolar concentrations) for the NZM2 line (BRAF wildtype) at 1 and 24 h.** The arrows indicate the protein of interest in blots where non-specific bands are also present.

ERK phosphorylation in response to CI-1040 was measured for NZM41, which is moderately resistant (IC_50_ = 200 nM). The phosphorylation status of MEK, which phosphorylates and activates ERK, was measured for comparison. Since expression of cyclin D1 has been reported to be down-regulated following MEK inhibition in cells with BRAF V600E mutations (Pritchard et al., 2007), expression of cyclin D1 was also measured, but there was no change in expression. ERK phosphorylation was inhibited at a CI-1040 concentration of 10 nM after 1 h but was comparatively unaffected after 24 h, even at 500 nM (Figure 3). This is in agreement with a report that sensitivity to a MEK inhibitor may decrease with exposure time (Friday et al., 2008). Interestingly, NZM41 showed evidence of CI-1040 resistance since MEK phosphorylation was increased following exposure to CI-1040 at 500 nM after 1 h and even at 50 nM after 24 h (Figure 3). The experiment was repeated with the NZM2 line, which is sensitive to CI-1040 (IC_50_ = 8.7 nM) and wildtype for *BRAF* and *NRAS*. ERK phosphorylation was highly sensitive to CI-1040 at both the 1-h and 24-h time points (Figure 4). No changes in MEK phosphorylation was observed but a decrease in cyclin D1 expression was apparent after 24 h.

## Discussion

New Zealand has a high incidence of melanoma and it was therefore of interest to compare the frequencies of activating BRAF and NRAS mutations in New Zealand-derived melanoma lines with published values. The BRAF V600E mutation frequently observed was found in this study at 30% (Table 2), lower than that reported by other groups (Davies et al., 2002; Houben et al., 2004; Edlundh-Rose et al., 2006; Thomas et al., 2007) while that for V600K was 5%. The mutation frequency for NRAS was 23% (Table 2), within the range reported by other groups (Davies et al., 2002; Houben et al., 2004; Edlundh-Rose et al., 2006; Thomas et al., 2007). The data in Figure 1 and Table 2 clearly show that cell lines with activating V600E and V600K mutations were generally sensitive to CI-1040, trametinib and vemurafenib inhibition. As shown in Table 2 the NZM28 cell line, which contained both G469A and L584F substitutions, was very sensitive to both inhibitors. SIFT algorithm analysis (Kumar et al., 2009) was undertaken to provide an indication of the effect of mutation, and predicted that the L584F mutation alters protein function, consistent with this effect. On the other hand NZM37, with a Thr600 insertion and NZM41, with a D594N substitution, were relatively insensitive to vemurafenib (Table 2), raising the question of why they might be selected for during melanoma development. The G469A mutation has been reported to have no enhancing effect on BRAF (Smalley and Flaherty, 2009) but it has been reported that kinase-dead BRAF mutations of D594 can have an indirect effect on tumor progression by enhancing CRAF activity (Heidorn et al., 2010). Several other studies have explored the relationship between mutation status and sensitivity to MEK inhibition for a variety of tumor types including melanoma, breast, ovarian, and lung cancers (Davies et al., 2002; Solit et al., 2006). In these studies, cell lines with *BRAF* mutations were very sensitive to MEK inhibition of cell growth while cell lines with *NRAS* mutations showed a range of sensitivities, in agreement with the present results.

It has been reported that either PI3K oncogenic mutations or deletion of PTEN reduces sensitivity of cells to MEK inhibitors (Wee et al., 2009). In this study, the NZM40 and NZM46 lines were found to have an activated mutated PI3K enzyme and the NZM6, NZM30, NZM34, and NZM43 lines were found to lack PTEN expression (Kim et al., 2012). However, there was no clear indication of altered sensitivity to CI-1040 among these cell lines. There are also reports that up-regulation of MEK can lead to reduced sensitivity of cells to MEK inhibitors (Friday et al., 2008). We investigated ERK phosphorylation in a number of melanoma lines (Figures 2–4). Although some evidence of loss of initial sensitivity in resistant lines was found (Figure 3) the pattern of phosphorylation results broadly followed that of the IC_50_ results.

In conclusion, we have assessed the responses of a series of 44 melanoma lines, generally of low passage number, to CI-1040, a prototypic MEK inhibitor, as well as to trametinib, a clinical MEK inhibitor and vemurafenib, a clinical BRAF inhibitor. We identified a sub-set of 16 lines (36%) with activating BRAF mutations (Figure 1) that showed sensitivity to both clinical inhibitors, supporting the hypothesis that a combination of both BRAF and MEK inhibitors might have advantages over either drug alone because of potentially synergistic inhibitory effects on signal transduction. We also identified a second sub-set of 10 cell lines (23%) that were resistant to vemurafenib but sensitive to a MEK inhibitor. Some but not all of these cell lines exhibited NRAS mutations, suggesting that some melanomas that are wildtype for both BRAF and NRAS may respond to trametinib, a MEK inhibitor. If this applies *in vivo*, then a proportion of melanoma patients whose disease is resistant to BRAF inhibitor therapy may respond to therapy with a MEK inhibitor.

### Conflict of interest statement

The authors declare that the research was conducted in the absence of any commercial or financial relationships that could be construed as a potential conflict of interest.
